# Primary Synovial Sarcoma of Lung: A Rare Tumor 

**Published:** 2016-04-24

**Authors:** Prince Raj, Parveen Kumar, Yogesh Kumar Sarin

**Affiliations:** Department of Pediatric Surgery, Maulana Azad Medical College, Delhi University, New Delhi

**Keywords:** Primary synovial sarcoma of lung, Recurrence, Immunohistochemistry

## Abstract

Synovial sarcoma of lung is a rare tumor with few case reports in literature. Though named synovial sarcoma due to its resemblance to synovium on light microscopy, it arises from mesenchymal tissue. Here, we present a case of synovial sarcoma of lung in a 7-year old boy, with main emphasis on difficulty faced in the management.

## CASE REPORT

A 7-year-old male child presented one year back with complaints of left sided chest pain. There was no associated history of cough, fever, respiratory distress or weight loss. On examination, there were diminished breath sounds on left hemithorax with basal crepitations. Chest radiograph showed homogenous opacity in left upper zone. CECT chest revealed ill-defined lesion in the left upper lobe compressing left main pulmonary artery and left sided pleural effusion causing collapse of underlying left lower lobe with mediastinal shift to right. CT guided biopsies were done twice, but each time it revealed necrotic material and tissue diagnosis could not be achieved.

At surgery, a large tumor arising from the upper lobe and adherent to the 2ndrib was found. Left upper pulmonary lobectomy with gross total excision of tumor was done. Histopathological examination of resected specimen revealed cellular tumor with oval shaped cells, moderate nuclear pleomorphism, and high nucleus to cytoplasmic ratio, vesicular chromatin, and mitotic figure of 10-11/hpf (Fig. 1a). Tumor cells were positive for vimentin, EMA, Bcl2 and CD 99 (Fig. 1b, 1c) and negative for calretinin, desmin, SMA, CD 34 and cytokeratin. The above features were consistent with synovial sarcoma.

**Figure:**
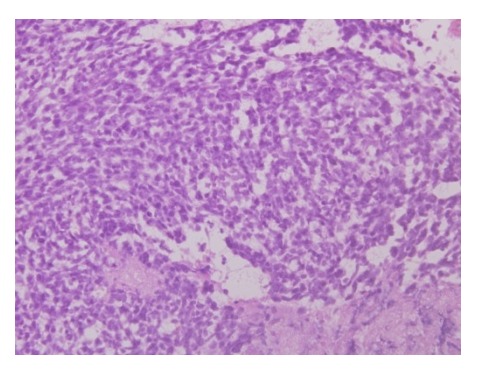
Figure 1a: Hematoxylin and Eosin staining showing tumor details.

**Figure:**
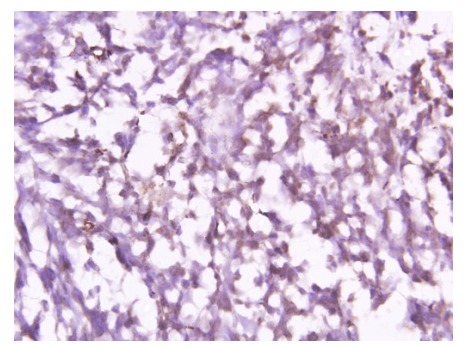
Figure 1b: Bcl2 positivity.

**Figure:**
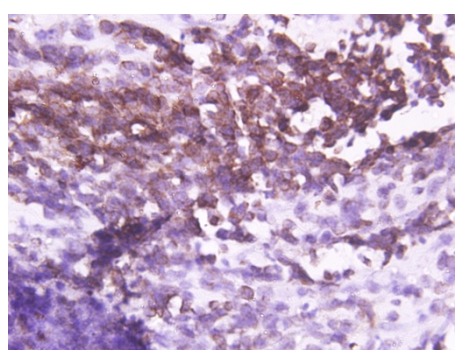
Figure 1c: CD99 positivity.

Postoperative PET-CT was negative with no tracer uptake. But as the histopathological margins were positive, the patient was planned for chemotherapy. He was started on ifosfamide, carboplatin, etoposide and epirubicin (ICE+ epirubicin). Three cycles of this chemotherapy regimen were administered, following which PET-CT was repeated. It revealed soft tissue density lesion in left 2nd intercostal space, with left sided pleural effusion suggestive of disease progression (Fig 2).

**Figure:**
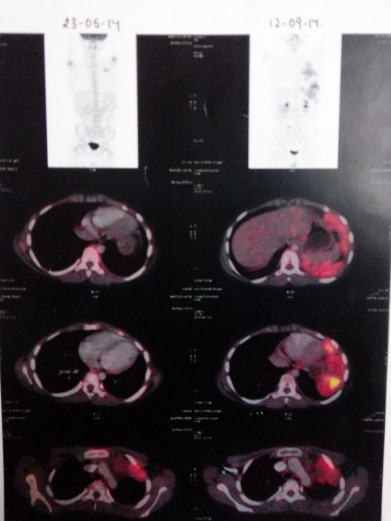
Figure 2:PET Scan showing residual tumor.

He was then subjected for radiotherapy. Intensity modulated radiotherapy (IMRT) was given five days a week for a total dose of 50.4Gy (28 fractions). PET-CT was done for response assessment, which showed metabolically active pleural-based soft tissue density lesion involving almost entire left pleura, suggestive of disease progression.

He was started on second line chemotherapy (docetaxel and gemcitabine) for total of 3 cycles. While on the third cycle, he developed swelling in the left back adjacent to the previous thoracotomy scar. Trucut biopsy from the swelling revealed the sheets of round to spindle tumor cells with moderate eosinophilic to clear cytoplasm, granular nuclear chromatin with inconspicuous nucleoli. Mitosis was 6-8/10 hpf. Findings were consistent with metastatic deposits.

CECT thorax was done and it showed a large mass 15x12x12 cm occupying whole of the left hemithorax with shifting of mediastinum to opposite side. The child developed respiratory distress and he was taken up for tumor resection. Intraoperatively, the tumor was densely adhered to the surrounding structures. Only piece-meal tumor debulking was possible. There was significant blood loss during the procedure and he had cardiac arrest twice during surgery. He was revived and shifted to ICU where he succumbed after few hours.

## DISCUSSION

Synovial sarcoma is the most common pediatric non-rhabdomyosarcoma soft tissue sarcoma (NRSTS) commonly affecting lower extremity followed by upper extremity. Other sites include head, neck and trunk. Primary synovial sarcoma of lung is a very rare entity and commonly presents as chest pain. Though termed erroneously due to its resemblance to synovium on histology, it is thought to arise from primitive pluripotent mesenchymal cells rather than the synovium.[1] It is an aggressive tumor with propensity to invade the adjacent tissue.[2]

Initial radiological investigations include chest X ray followed by CECT thorax. More than 75% of the cases show well-delineated border on initial CXR.[3] Ipsilateral pleural effusion is common, which was present in our case too. Management of such tumor is difficult as these tumors are poorly sensitive to chemotherapy. Surgical resection whenever feasible is the treatment of choice.

On histopathological examination (HPE), it needs to be differentiated from fibrosarcoma. Immunohistochemistry (IHC) is needed to differentiate it from other sarcomas. Synovial sarcoma is positive for cytokeratin, ema, bcl-2, vimentin and negative for s-100, desmin.[4-6] In our case, it was positive for vimentin, ema, cd 99 and bcl2 and negative for calretinin, desmin, sma, cd 34 and cytokeratin. [7, 8]

Prognosis depends on the size of tumor and the age of patient. Other factors are male gender, extensive tumor necrosis, high grade, large number of mitotic figures (>10/hpf) and syt-ssx1 variant. [9] SYT-SSX translocation is characteristic of synovial sarcoma, found in more than two third patients. SYT-SSX1 variant is associated with more aggressive phenotype and more tumor cell proliferation. It could not be done at our centre due to non availability of resources. Most of these poor prognostic factors including age, male gender, high grade, necrotic tissue and high mitotic rate were present in our case too. For children 0 to 16 year old and tumors less than 5 cm in size, 5-yearoverall survival (OS) is 71 to 88 %, and in this group, addition of chemotherapy did not improve survival. In patients with tumor greater than 5 cm that are deep and invasive and without metastasis, OS is 50 to 75 % and chemotherapy responsiveness is 50 to 60 %. Radiotherapy does have role in this disease and is recommended after marginal resection or before anticipated marginal resection. Standard protocol for adjunct chemotherapy is lacking due to its rarity and poor chemo-sensitivity.

To conclude, primary synovial sarcoma of lung is a very rare tumor, which is highly aggressive with poor prognosis. Diagnosis needs combined efforts with radiology, histology, immunochemistry and molecular studies. The most appropriate management is surgical excision with negative margins, whenever feasible. Adjuvant chemotherapy and radiotherapy have limited role. Due to high rates of recurrence, a very close follow up is needed.

## Footnotes

**Source of Support:** Nil

**Conflict of Interest:** None declared

